# Photography of Radioscopic Images

**Published:** 1918-05

**Authors:** 


**Photography of Radioscopic Images.** Egon Hartung, quoted in Arch. d'Electricite Med., Oct. 1917, advises the use of a screen without lead glass, emitting a bluish fluorescence, special photographic objectives and a localizing cylinder of lead, impermeable to the rays. A very fine focus and ultrasensitive photographic plates are necessary, the latter such as are used for astronomic observations. He has adapted the apparatus to cinematography.

